# A Nanoparticle, Fluorescence-Based Rinse for Caries Activity Assessment, an in Vitro Study

**DOI:** 10.1016/j.identj.2025.02.003

**Published:** 2025-05-06

**Authors:** Bruna Leticia Vessoni Menoncin, Wendy Bloembergen, Nathan Jones, Adam Hoxie, Matthew Finkelman, Andrea Ferreira Zandona

**Affiliations:** a Department of Comprehensive Care, Tufts University School of Dental Medicine, Boston, Massachusetts, USA; b GreenMark Biomedical Inc., Ann Arbor, Michigan, USA; c Division of Diagnostic Sciences, Adams School of Dentistry, Chapel Hill, North Carolina, USA; d Department of Public Health and Community Service, Tufts University School of Dental Medicine, Boston, Massachusetts, USA; e Division of Restorative and Prosthetic Sciences, The Ohio State University College of Dentistry, Columbus, Ohio, USA

**Keywords:** Dental caries activity tests, Nanoparticles, Caries detection, sensitivity, fluorescence

## Abstract

**Objectives:**

This in vitro study aimed to compare the agreement of a starch-based nanoparticle fluorescence rinse (FR) (LumiCare™ Caries Detection Rinse, GreenMark Biomedical Inc.) in the assessment of caries activity of initial smooth surface carious lesions with the International Caries Classification and Management System (ICCMS) Lesion Activity Assessment.

**Methods:**

A convenience sample of permanent extracted human teeth (N = 57) were scored by visual assessment (VA) using the ICCMS activity criteria by 2 calibrated examiners. VA categories included: sound, inactive and active. For fluorescence assessment (FA), each tooth was fully immersed in FR for 30 seconds, rinsed with water for 10 seconds, compressed air dried and examined under an orange filter while illuminating with a curing light. Fluorescence was assessed as no fluorescence or fluorescence. Both VA and FA were repeated after at least 24 hours and Kappa was calculated for inter- and intra-examiner agreement.

**Results:**

Based on VA, 12 teeth (21%) were classified into the active category, while 45 teeth (79%) were classified into the sound (n = 20) or inactive (n = 25) categories. When combining the latter 2 categories, VA and FA’s classifications were concordant in 100% of cases (all 12 teeth in VA’s active category exhibited fluorescence, and all 45 teeth in VA’s sound/inactive category did not exhibit fluorescence according to FA; Kappa = 1).

**Conclusion:**

FA with FR demonstrated perfect agreement with VA using ICCMS when comparing active versus inactive and/or sound surfaces suggesting it has potential as an objective indicator of caries activity.

**Clinical significance:**

A fluorescence rinse could improve clinical detection of smooth surface caries activity and **c**ould easily be integrated as part of the dental hygiene appointment.

## Introduction

Dental caries is the most prevalent chronic disease in the world.^1^ Carious lesions are usually detected by visual^2^ and radiographic complementary exams.^3^ The visual-tactile exam is a simple technique with no additional costs. Shortcomings are related to its subjectivity^4^; low sensitivity, particularly for early noncavitated carious lesions; and potential for iatrogenic damage if a tactile exam with a sharp metal dental explorer is used to assess hardness.^5^ Radiographs expose the patient to radiation and have a low sensitivity for initial lesions particularly on occlusal surfaces and are of limited use on free smooth surfaces.^6^

For treatment decision making, detection of caries lesions must be associated with assessment of caries activity.^7^ An active caries lesion is considered to be progressive; that is, the lesion continues to demineralize.^8^ A lesion that has stopped further progression is referred to as an inactive/arrested caries lesion. In sum, active lesions are more likely to progress than inactive/arrested lesions.^9^ However, clinical assessment of caries activity is fraught with subjectivity. Although several technology-based methods have been developed aiming to improve accuracy for caries detection,^10^^,^^11^ until recently few have been developed as potential tools for caries activity assessment. In theory, any technology-based device that provides an objective assessment of a carious lesion could determine caries activity by assessing changes over time, which is the true gold standard measure of caries activity. However, in clinical practice, a point-of-care diagnostic method with reliable assessment of caries activity would be advantageous for treatment-decision making allowing implementation of more targeted prevention protocols and better monitoring of outcomes.^11^ Visual assessment using the International Caries Classification and Management System (ICCMS) Lesion Activity Assessment, has been shown to be predictive of caries progression in longitudinal studies.^12^^,^^13^ It was therefore selected as the reference test in this study as it is a validated and accepted assessment of caries lesion activity in diagnostic test evaluations.^14^

LumiCare™ Caries Detection Rinse (GreenMark Biomedical Inc.) (FR) an aqueous oral rinse that uses proprietary nanoparticles to illuminate early carious lesions in teeth using a simple dental curing light, recently received FDA clearance to assist the dental professional in the visualization of dental caries. The rinse contains patented fluorescent sub-micron starch particles that have been functionalized to target and adhere to the subsurface of early-stage carious lesions after penetrating through surface porosities.^15^ The cationic starch particles in the LumiCare™ rinse (FR) are attracted through electrostatic driving forces into the patient’s porous enamel. These particles are activated by blue light and visualized when viewed through an orange-colored lens. Based on the principle that the FR would only penetrate enamel surface porosities, we hypothesize that this rinse would be able to assess activity status of initial smooth surface carious lesions. In previous work, FR was evaluated for *in vitro* diagnostic accuracy in detecting occlusal caries (limited to active appearing lesions), finding strong intra- and inter-user reliability and high sensitivity and specificity when compared to gold-standard histology.^16^ The previous work evaluated the diagnostic accuracy for caries detection, for which histology is the gold standard, not caries activity. Although it is known that remineralization during the inactivation of carious lesions tends to create a hard surface layer on the outermost part of enamel^8^^,^^17^^,^^18^ with an increase in mineral density of the surface layer for inactive lesions compared to active demineralization sites^19^ there is no gold standard for caries activity assessment in vitro, thus a validated clinical criteria was used. An additional study found that analyzing fluorescence images taken following application of FR with a convolutional neural network AI model showed promise for caries detection, severity assessment, and lesion surface porosity as an indicator of activity under the International Caries Detection and Assessment System (ICDAS) framework.^20^ The current study now focuses on the assessment of caries activity, evaluating inactive and active smooth surface non-cavitated lesions and sound surfaces, comparing FA to an ICCMS visual tactile activity assessment.

The aim of this in vitro study was to compare the agreement of FA (index test) to assess caries activity of initial smooth surfaces carious lesions with ICCMS Lesion Activity Assessment (reference test).

## Materials and methods

A convenience sample of N = 59 extracted permanent human teeth was used in the study. Selected teeth were either sound or had non-cavitated (ICDAS 1-2) smooth coronal surface carious lesions and were free of developmental defects. They were stored in 0.2% thymol solution at 4°C.

### Examiner calibration for clinical visual assessments

Two experienced dentists reviewed the online ICDAS Program (ICDAS Calibration for International Caries Classification and Management System ICCMS–™ - https://www.iccms-web.com). For training, examiners independently assessed a set of 31 images of active and inactive lesions and 20 teeth using the ICCMS criteria and the FA criteria (yes/no for fluorescence) with at least a 24h interval between exams. They then reviewed the scores together and discussed any disagreements to reach a consensus. Examiners were considered calibrated when weighted Kappa scores for activity were 0.61 or higher for inter-examiner agreement and 0.81 or higher for intra-examiner agreement.

### ICCMS lesion activity assessment of samples (VA)

**Fig. 1:**
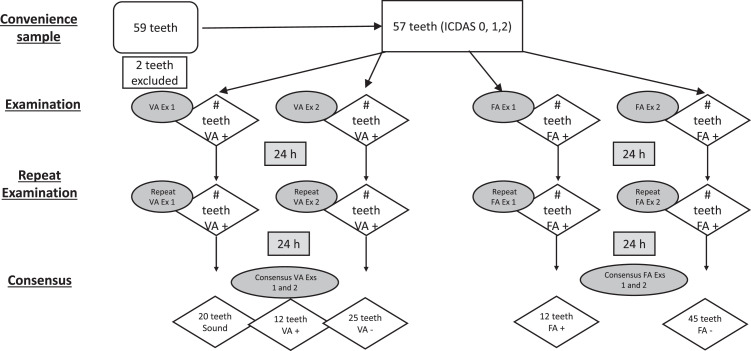
Flowchart illustrating the selection process of the 59 teeth.

For this reference test, teeth were categorized based on ICCMS assessment criteria as: sound (no evidence of caries); inactive (whitish, brownish or black enamel, shiny and smooth when the tip of the probe is moved gently across the surface); and active (whitish/yellowish opaque enamel surface with loss of luster, feels rough when the tip of the probe is moved gently across the surface). Teeth were examined wet, then dried for 5 seconds using the criteria in Table 1. In the case of any disagreements between the 2 examiners a consensus was determined by re-examining the teeth together (Figure 1).

**Table 1:** Examples of lesion types by visual and fluorescence assessment criteria.

Active lesions		Inactive lesions		Sound surfaces	
visual	Fluorescence	visual	Fluorescence	visual	Fluoresecence
whitish/yellowish opaque enamel with loss of luster, rough with the probe across the surfaces	Yes	whitish, brownish or black enamel, shiny and smooth when the tip of the probe was moved gently across the surface	No	No evidence of caries	No

### Fluorescence assessment of samples (FA)

Extracted teeth were individually dipped into the FR solution for 30 seconds, lightly swirled to simulate swishing, immersed in DI water for 10 seconds with light swirling, then dried with a compressed air syringe. The teeth were kept in a humid environment, but not immersed in water until examination (no more than 20 minutes after exposure to the rinse). An image of the tooth was obtained with a microscope and an orange protective filter (SZX16, OLYMPUS using a Uvex Ultra Spec 2000 (Honeywell, Charlotte, NC, USA), while illuminating the tooth with a Dental Power LED curing light (Aphrodite, USA), held at a distance of 1-2 inches (2.5-5 cm). To minimize bias a 24 h interval between the examinations was done (Figure 1). The 24 h interval was a convenience interval that has been used in other studies.^21^, ^22^, ^23^ Teeth were categorized as positive or negative for fluorescence (FA).

To assess intra-examiner agreement, examiners repeated both exams independently with at least a 24-hour interval.

### Data analysis

Descriptive statistics were calculated. Intra- and inter-examiner agreement was calculated via Cohen`s Kappa. To determine inter-examiner agreement, data from both readings by each examiner were used. Two-by-two contingency tables summarizing the results of FA compared to VA were created based on the consensus agreement between the 2 examiners. The agreement of FA and VA was evaluated using positive percent agreement (PPA, which is analogous to sensitivity) and negative percent agreement (NPA, which is analogous to specificity). Cohen’s Kappa was also calculated for each comparison. This metric can range from -1 to 1 and can be interpreted as: <0.2 as none to slight; 0.21-0.4 as fair; 0.41-0.6 as moderate; 0.61-0.8 as substantial; and 0.81-1.0 as almost perfect agreement. Tests of association were conducted using Fisher’s exact test to determine whether the classifications of FA and VA were significantly associated with each other. Analyses were performed to investigate the agreement of the methods in determining (1) lesion presence or absence and (2) lesion activity (when lesion was present). Thus, the following groupings were established by visual assessment: sound versus lesion (active and inactive); active lesion versus inactive lesion (excluding sound based on VA); and sound and inactive lesion versus active lesion. The significance level was set at α = .05. SPSS v. 28 (IBM Corp., Armonk, NY, USA) was used in the analysis.

## Results

Two of the 59 teeth were excluded (as upon further examination they did not meet the inclusion criteria) yielding a final sample size of N = 57 for analysis. There were 18 teeth that examiners did not agree on activity status based on visual assessment and 16 teeth based on fluorescence assessment. Examiners reevaluated the teeth together for VA, and 24h later for FA to reach a consensus. After re-evaluation, 12 teeth (21%) were classified into the active category, while 45 teeth (79%) were classified into the sound or inactive categories.

Table 2 presents Cohen’s Kappa values for intra- and inter-examiner agreement. All intra-examiner values for VA and FA fell into the substantial or almost perfect agreement categories, while the inter-examiner values for VA fell into the moderate agreement category and FA fell into the substantial agreement category.

**Table 2:** Intra- and inter-examiner reliability (Cohen's Kappa) values and standard deviation for visual assessment (VA) and fluorescence assessment (FA) (N = 57 teeth).

	Intra-examiner reliability		Inter-examiner reliability
	Examiner 1	Examiner 2	Examiners 1 and 2
VA	0.84 ± 0.12	0.72 ± 0.16	0.52 ± 0.12
FA	0.81 ± 0.13	0.75 ± 0.14	0.69 ± 0.11

Table 3 presents the cross-tabulation between the VA and FA classifications. When considering active versus inactive lesions, excluding sound (middle panel), all inactive lesions based on VA exhibited no fluorescence based on FA, and all active lesions based on VA exhibited fluorescence based on FA. The association between VA and FA was significant (p<.001). The Kappa statistic was 1, and the PPA and NPA were both 100%. When considering sound teeth and inactive lesions versus active lesions (bottom panel), all sound teeth and inactive lesions based on VA exhibited no fluorescence based on FA, whereas all active lesions based on VA exhibited fluorescence based on FA. As in the middle panel, the association between VA and FA was significant (*P* < .001), the Kappa statistic was 1, and the PPA and NPA were 100%. When considering sound versus any lesion (active and inactive combined) for VA (top panel of Table 3), the association between VA and FA was statistically significant (*P* = .005). The Kappa statistic was 0.252, while the PPA and NPA were 32% and 100%, respectively, using VA as a reference.

**Table 3:** Concordance between visual examination and fluorescence assessment.

		Fluorescence Assessment		Kappa	PPA (%)	NPA (%)	OR	P*
		No Fluorescence	Fluorescence					
Visual Exam (reference)	Sound	20	0	0.252	32	100	N/A	.005
	Lesion	25	12					
	Inactive	25	0	1	100	100	N/A	<.001
	Active**	0	12					
	Sound + inactive	45	0	1	100	100	N/A	<.001
	Active⁎⁎⁎	0	12					

⁎ P-values were obtained from Fisher`s exact test.

⁎⁎ The 20 teeth classified by VA as sound were not included in the analysis.

⁎⁎⁎ The 20 teeth classified by VA as sound were included in the analysis.

## Discussion

Clinical indicators used to assess caries activity at the lesion level include visual appearance, tactile feeling, potential for plaque accumulation and, for cervical lesions, the gingival health status.^5^ The visual and tactile assessment of all these characteristics to determine caries activity is subjective. Technologies have been developed to help professionals^11^ with more objective tools. There is limited indication that the Quantitative Light-induced Fluorescence (QLF) technique can quantify enamel de- and remineralization for the early assessment of caries activity^24^, ^25^, ^26^, ^27^ either by assessing rapid changes in fluorescence loss during dehydration or by assessment of red fluorescence.^26^^,^^28^^,^^29^ However, for various reasons, these technologies also have not been widely adopted clinically. A validated objective diagnostic method to assess caries activity at the point of care remains an unmet need.

Based on the principle that the FR would be able to penetrate only the surface of active lesions due to the microchannels present, as presented in Jones et al.,^15^ analysis in this study was initially performed for sound and inactive lesions (versus active lesions). In theory the rinse should not penetrate either sound or inactive surfaces. Additionally, in practice neither sound nor non-cavitated inactive caries lesions as those selected in this study require clinical intervention. Results showed perfect agreement of positive FR fluorescence and lesions categorized as active by VA, as well as perfect agreement of no fluorescence with inactive lesions/sound surfaces by VA. In an analysis of lesions only (active and inactive), excluding sound surfaces, similarly there was perfect agreement of positive fluorescence and active lesions and perfect agreement of negative fluorescence and inactive lesions. However, in an analysis of any lesion (active and inactive) versus sound surface, positive agreement of FR fluorescence and any lesion was only 32%, and Kappa was only 0.25, consistent with fair agreement. This is to be expected considering the FR mechanism of action, i.e., penetration of microporosities that are only present in active lesions, which would therefore theoretically only fluorescently illuminate active lesions. In terms of reproducibility, it can be noted that the intra- and inter-examiner reliability for FA in this study was comparable to previously published data which was limited to occlusal surfaces (0.80, 0.94, by examiner, and 0.74, respectively).^16^ Differences in surface type (occlusal versus smooth) could affect visibility of fluorescence. The previous study also included more advanced lesions including cavitated lesions, which theoretically should be easier to detect.

To summarize, these results suggest that FR has potential to be used to objectively differentiate whether a caries lesion is active or inactive. This is a critical component of appropriate caries diagnosis to enable correct clinical decision-making for management of carious lesions.^8^ Distinguishing caries activity has the possibility to shift from a restorative treatment to a prevention-driven approach and engage patients in treatment planning.^11^ Although ICCMS Lesion Activity Assessment is used for research purposes, it is not commonly used in the primary setting due to various obstacles.^30^ The FR could additionally be useful in detection of active (only) lesions, perhaps for screening purposes. FR certainly fulfills the goals of a caries detection method as stated by Pitts & Stamm^31^ by detecting lesions from the initial white spot lesion stage.

In this study, ICCMS was selected as the reference test because it has been validated as an assessment of caries lesion activity in diagnostic test evaluations.^14^ That it can aid monitoring, particularly in caries clinical trials,^32^ supports its validity as an assessment of activity. Limitations of the current study include evaluation of smooth surface lesions only and the in vitro nature of the study. Clinically, the presence of saliva, biofilm, calculus and surface defects, like microcracks or enamel hypomineralization may impact the penetration of the rinse.

Although FA had perfect agreement with VA in this *in vitro* study, further studies are needed to determine clinical performance. Longitudinal clinical studies will be critical to determine reliability of FR to identify lesions that are truly active, meaning ones that progress and continue to demineralize. However, even longitudinal clinical studies will be fraught with challenges, as active, progressing carious lesions oscillate between demineralization and remineralization cycles and may become inactive during the study.^33^

Other devices currently in development as tools for activity assessment include Calcivis, (Edinburgh, UK) a technology-based device that aims to assist caries activity assessment through luminescence.^34^ The system is based on delivery of a photoprotein to the enamel surface, which in the presence of free calcium (from active carious lesions) luminesces. BlueCheck (Incisive Technologies, Melbourne, Australia) is a blue liquid that has an affinity for porous enamel, thus easily identifying areas on the tooth that require clinical attention and informing the patient of the need for better brushing.^35^

## Conclusion

FA with FR demonstrated perfect agreement with VA using ICCMS when comparing active versus inactive and/or sound surfaces suggesting it has potential as an objective indicator of caries activity. Further studies are warranted to determine performance and validity in the clinical setting.

## Conflict of interest

The authors declare the following financial interests/personal relationships which may be considered as potential competing interests:

Dr. Zandona is a consultant for Greenmark. Drs. Wendy Bloembergen and Nathan Jones are employees of this company. The other authors have no conflicts of interest to declare.
